# Undergraduate rheumatology teaching in the UK: a survey of current practice by teachers and students

**DOI:** 10.1093/rap/rkae112

**Published:** 2024-09-13

**Authors:** Pippa Watson, David Hanna, Sophia M Wakefield, David Coady, Donna Andrew, Daisy Southam, Richard J Wakefield

**Affiliations:** Department of Rheumatology, Manchester University NHS Foundation Trust, Manchester, UK; School of Medicine, Newcastle University Faculty of Medical Sciences, Newcastle upon Tyne, UK; School of Medicine, Cardiff University, Cardiff, UK; Department of Rheumatology, South Tyneside and Sunderland NHS Foundation Trust, University of Sunderland, Sunderland, UK; Healthcare Professional Training and Education Manager, Versus Arthritis, Chesterfield, UK; Head of Education, British Society for Rheumatology, London, UK; Department of Rheumatology, University of Leeds, Leeds Teaching Hospitals Trust, Leeds, UK

**Keywords:** rheumatology, curriculum, undergraduate, training, education, students

## Abstract

**Objectives:**

The last major UK survey of medical undergraduate rheumatology teaching was 25 years ago. This study aimed to describe current teaching practice, the perceptions of teachers and students and their engagement with Versus Arthritis teaching resources and future challenges and opportunities.

**Methods:**

Electronic surveys were distributed by e-mail and/or social media to relevant teachers and students identified within all 37 UK medical schools.

**Results:**

A total of 34/37 (91%) teacher and 30/37 (81%) student surveys were returned. Compared with the last survey, the proportion of schools delivering rheumatology-identifiable teaching has fallen from 100% to 86% and the mean number of teaching days from 30 to 10. Rheumatology teaching is now more dispersed throughout the curriculum. Students preferred active learning methods such as simulation and expert patient teaching, while teachers preferred small-group teaching, online learning and lectures. The Versus Arthritis resources appeared underutilized by students but were considered useful. Most students thought rheumatology careers were not promoted within their medical school.

**Conclusion:**

A decrease in dedicated rheumatology teaching time was noted since the last survey 25 years ago. Greater promotion of rheumatology as a speciality and future career is required to maintain its professional identity and prevent marginalization.

Key messages

- Curricula time dedicated to medical undergraduate rheumatology teaching is less than it was 25 years ago.
- Educators should deliver teaching in line with student preferences, including providing opportunities for active learning.
- Signposting to Versus Arthritis resources and rheumatology career guidance should be considered.

## Introduction

A decrease in dedicated rheumatology teaching time was noted since the last survey 25 years ago. Greater promotion of rheumatology as a speciality and future career is required to maintain its professional identity and prevent marginalization.

Musculoskeletal (MSK) complaints account for approximately one-third of primary care consultations [1] and are commonly encountered on hospital wards and in emergency departments. It is important therefore that all doctors know how to identify and manage them. It is now almost 25 years since Kay *et al*. [2], in conjunction with the British Society for Rheumatology (BSR) and Arthritis Research Campaign (ARC), surveyed UK medical schools about their rheumatology undergraduate programs. Since then, the medical education landscape has significantly changed, with increasing medical school numbers (from 26 to 37), rising student intakes, the introduction of new technologies and changes in teaching pedagogies and student expectations, not to mention the global COVID-19 pandemic.

The General Medical Council [3] sets out competencies that need to be achieved prior to graduation. However, what and how a medical subject is taught is largely dependent on the different educational philosophies and priorities of individual medical schools [4, 5]. This allows for wide potential variations in teaching practices across the UK. The impact of a new UK medical licensing assessment (UKMLA) [6] requiring all UK medical students to sit for the same knowledge-based exam is yet to be seen.

The overarching aim of this study was to capture contemporaneous data about rheumatology undergraduate teaching by UK medical schools. We explored two perspectives: the teachers—those who organize and deliver the teaching programs—and the students—those who receive the teaching.

The specific objectives were, first, to determine when, where and how rheumatology-based teaching is included in the curriculum of each UK medical school; second, to gain an understanding of how students want to learn; third, whether they engage with rheumatology-based resources such as those provided by Versus Arthritis; and finally, to identify opportunities and challenges for future rheumatology education.

## Methods

An initial literature search identified relevant previous work including the surveys by Kay *et al*. [2] and Jones *et al*. [7]. A small working group (three consultant rheumatologists with an interest in medical education, two medical students (then year 2 and year 4) and representatives from BSR Education and Versus Arthritis was formed. Teacher and student questionnaires were developed in parallel.

A web-based SurveyMonkey link was e-mailed via the BSR to named teachers representing all 37 UK medical schools on May 21. These were identified from existing lists held by the BSR and Versus Arthritis, known working group contacts, medical school websites and by word of mouth/personal recommendation. We aimed to reach the undergraduate rheumatology education lead at each medical school. The teacher survey included 44 open and closed questions, using previous survey themes and additional areas such as the impact of COVID. Reminders were sent out after 2 and 4 weeks.

**Figure 1.**
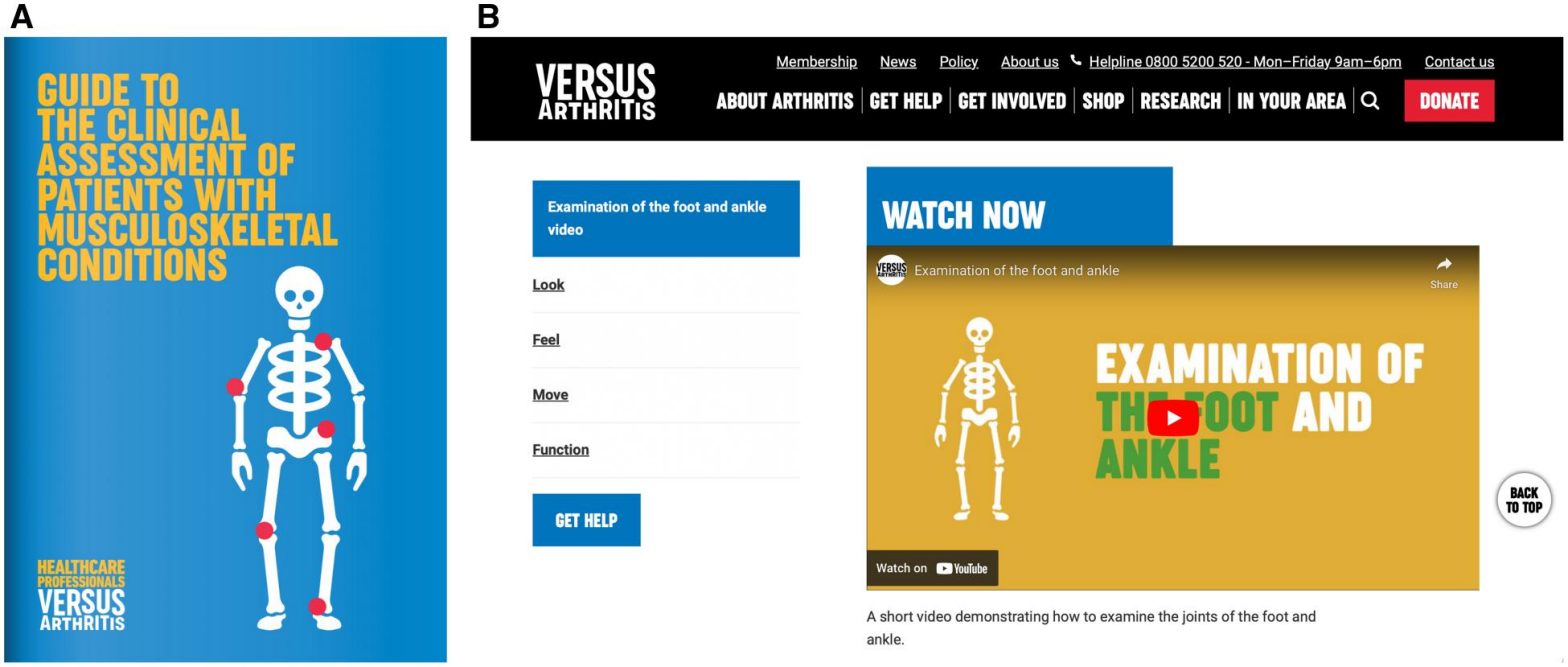
Versus Arthritis training resources. **(A)** MSK clinical assessment flipbook [8]. **(B)** YouTube videos cover screening and regional MSK examinations with simulated and real patients [9]

Due to General Data Protection Regulation, obtaining the names of students was more difficult, as there was no available database. Initial e-mail contact was made to each student medical society for all schools. An initial low response rate prompted a ‘snowballing’ contact technique via social media, including SMS, WhatsApp, Facebook Messenger and Instagram. After verbal agreement, students were contacted by e-mail and asked to complete an electronic questionnaire. While similar to the teacher survey, it also provided an additional emphasis on students’ own reflections of teaching methods and clinical placements and their use of available teaching resources. In this latter respect, reference was made to the MSK handbook and examination videos provided by the Versus Arthritis website (Fig. 1).

## Results

From the 37 medical schools, 34 (92%) teacher surveys and 30 (81%) student surveys were returned. One-third of student responders were in their pre-clinical years. Not all respondents answered all the questions.

### Exposure to rheumatology teaching

Eighty-six percent of schools reported rheumatology-related education in their curriculum, with a mean duration of 10 days (range 3–30). Pre-clinical (years 1 and 2) course content varied and included generic MSK lectures within anatomy and physiology modules, specific lectures on arthritic conditions and problem-based learning tutorials. A small number of medical schools delivered MSK examination skills in the first 2 years.

Ninety-seven percent of students identified general MSK training within their curriculum, but only 57% identified teaching as rheumatology based. For most schools, clinical rheumatology instruction took place in year 4 (50%) compared with 23% in year 3 and 27% in year 5.

The delivery of clinical placements differed across medical schools. Eleven of 27 schools (40.7%) delivered rheumatology as a block, 9 schools (33%) spread teaching over their clinical years, 2 schools delivered it in a term and 1 school delivered it throughout 1 year, with the remainder as a combination. In most schools [22/27 (82%)], rheumatology was delivered alongside another specialty teaching, most commonly orthopaedics (*n* = 19), care of the elderly (*n* = 7), general practice (*n* = 6), rehabilitation (*n* = 4) and dermatology (*n* = 4). Other linked specialties included palliative care and infectious diseases. It was estimated that 25% of rheumatology training was delivered in the community. Eighty-two percent of teachers reported formal student interaction with members of an multidisciplinary team during their clinical placement, including specialist nurses, physiotherapists, occupational therapists and podiatrists.

### Teaching methods and use of resources

Teaching methods varied across medical schools. Teachers ranked their most commonly used methods: small-group teaching, online learning and lectures. In contrast, students ranked their preferred teaching methods as expert patient teaching and teaching objective structured clinical examination (OSCE) sessions. Small-group learning appeared valued by both. Lectures, online learning and simulation were least favoured by students [Table 1].

**Table 1.** Hierarchy of teaching methods most commonly used and teaching methods reported most useful by students

Most used methods (as reported by teachers)	Most effective methods (as reported by students)
Lectures	Small group
Small group	Expert patient
Online	Teaching OSCEs
Expert patient	Lectures
OSCEs	Simulation
Simulation	Online

The Versus Arthritis resources were utilized by 80% of clinical rheumatology teachers. In contrast, only 17% of students reported being aware of them or did not use them. Those students who used them reported improvements in their knowledge and examination skills and appreciated the ability to revisit them and learn at their own pace at home.

### Assessment

All teacher and student respondents acknowledged that rheumatology subjects were formally assessed within their programs. The teachers had varied perceptions and knowledge of the impact of the new UKMLA on the rheumatology curriculum; 26% (*n* = 7) believed there would be a significant impact on the curriculum, 23% (*n* = 6) believed there would be limited or no impact and the remaining 50% (*n* = 13) were unsure.

### Effect of COVID

Due to the COVID-19 pandemic, 50% of medical schools suspended clinical placements at some point while those that continued did so with heavy modifications. Rheumatology teachers reported a 49% average decrease in clinical patient contact for students. Students reported a 41% average decrease in patient contact.

In response to this decrease in clinical time, medical schools/clinical teachers increased their use of both synchronous [lectures, tutorials (e.g. via Teams, Zoom etc)] and asynchronous online learning (Virtual Learning Environment and external website resources). In the clinic setting, 15 schools reported engaging their students in telephone clinics, while 9 had students participating in video consultations, either from home or the clinic. Only nine schools reported having access to these resources prior to the pandemic.

### Barriers to learning

From the teacher survey, the major barriers to learning included pressures to deliver clinical work, the high volume of students with competition from other learners and a shortage of administrative support. In addition, the teachers also reported a general low morale of National Health Service (NHS) staff, which worsened during COVID, few teaching-dedicated staff and a lack of recognition of academic time.

### Promotion of rheumatology as a career

Teachers reported non-teaching opportunities for students to explore rheumatology as a career option. These included career events, ‘taster days’, introduction to learning resources (e.g. those from Versus Arthritis) and opportunities to work on quality improvement projects. In contrast, 86% of students reported little to no career promotion of rheumatology as a specialty at their schools. The delivery of rheumatology relatively late in medical training (25% in year 5) may also impact on initial career choices, as many students have already formed ideas of what they want to do by then and have begun to make their foundation year application choices.

## Discussion

This survey of UK undergraduate rheumatology education provides contemporaneous insights from both the perspective of the teacher and, importantly, the students themselves. This is in contrast to the last study by Kay *et al*. [2], which was 25 years ago and sampled only faculty members. The high response rate of 92% of teachers and 81% of students may reflect the perceived importance of teaching in rheumatology or a more targeted sampling strategy.

Only 86% of medical schools currently provide dedicated time for rheumatology training in contrast to all 26 medical schools in 1997 [2]. In addition, the amount of dedicated rheumatology teaching time decreased from 30 to 10 days [2]. From the student survey, only 57% of respondents were able to identify specific rheumatology teaching, although 97% acknowledged general MSK teaching.

The apparent reduction in exposure to rheumatology-specific teaching is important because not only will future doctors be less equipped to manage rheumatology-specific conditions, e.g. inflammatory arthritis, the reduced exposure to rheumatology might also affect the number of future trainees applying for positions, which has been noted to have decreased in recent years. Watson and Gaffney [10] previously suggested that increased exposure to rheumatology as an undergraduate may heighten interest in the specialty. As well as a reduction in the total time dedicated to rheumatology, there was also a tendency towards more dispersed and integrated teaching. While having some advantages with respect to timetabling and learning about multidisciplinary working, it may also have the effect of diminishing the identity of ‘rheumatology’ as a stand-alone specialty.

The new UKMLA curriculum [6] may influence what medical schools decide to teach in the future, with teachers uncertain about the future impact of this on their own curriculum. It remains encouraging, however, that several rheumatology-related conditions and ‘clinical presentations’ are included, although omissions exist, most notably ‘giant cell arteritis’. It is perhaps also noteworthy that the outcomes are listed under ‘musculoskeletal’ rather than ‘rheumatology’. This likely highlights the increasing multidisciplinary nature of the speciality and current service delivery, but curriculum developers in medical schools should be mindful not to remove the ‘rheumatology’ specialization altogether.

COVID had a significant effect on time in placement, with both teachers and students reporting a significant reduction in time in placement from 41 to 49%, in line with reports [11]. The pandemic also likely compounded some of the long-standing barriers to teaching students, including high workload and lack of time.

Noticeable differences between the teacher and student surveys related to teaching delivery methods and the promotion of careers and resources. Small-group teaching was valued by both teachers and students, but the teachers also liked lectures and e-learning, while students preferred ‘active learning’ sessions with ‘expert patients’ and ‘teaching OSCEs’. While teachers thought they promote careers and directed students to resources such as those from Versus Arthritis, this was not recognized by all students. The Versus Arthritis resources, although reported as being very useful by students who used them, perhaps could be more widely signposted and shared.

One of the main limitations of our student survey was that one-third of students were in their pre-clinical years and may have had limited knowledge of the later clinical program, although they were encouraged to ask others. For the teacher survey, while we endeavoured to identify the rheumatology education lead, it was not possible to be certain that we reached the most appropriate person, as there was often no single person who oversaw the whole curriculum. We also did not consider the different sizes of medical schools and that many new ones are opening. Our study was also prior to the announcement in the NHS workforce plan about further increases in student numbers [12].

In conclusion, since the last major survey 25 years ago, the trend appears to be a reduction in rheumatology-specific specialty exposure and more dispersed and integrated teaching delivered within the undergraduate curriculum. The effect of these changes on competence and performance, in addition to the introduction of the new UKMLA, is currently unknown and warrants further study. There appears to be a paucity of rheumatology-specific career promotion activities and signposting to Versus Arthritis resources. We believe that the whole rheumatology community needs to actively and collectively engage in shaping the future of rheumatology education.

## Data Availability

Data are available upon request.
